# Effect of Kinesio Tape Application on Calf Pain and Ankle Range of Motion in Duathletes

**DOI:** 10.2478/hukin-2013-0033

**Published:** 2013-07-05

**Authors:** Rafael Merino-Marban, Daniel Mayorga-Vega, Emilio Fernandez-Rodriguez

**Affiliations:** 1 Physical Education, College of Educational Science, University of Malaga, Spain.; 2 Department of Physical Education and Sport, University of Granada, Spain.

**Keywords:** Kinesio taping, Numerical Pain Rating Scale, ankle dorsiflexion, duathlon competition

## Abstract

The purpose of this study was to examine the effect of the kinesio tape immediately after its application and after a duathlon competition on calf pain and the ankle range of motion in duathletes. A sample of 28 duathletes (age 29.11 ± 10.35 years; body height 172.57 ± 6.17 cm; body mass 66.63 ± 9.01 kg; body mass index 22.29 ± 2.00 kg/m ^2^ ) were recruited from the competitors in a duathlon sprint. The Numerical Pain Rating Scale and ankle dorsiflexion range of motion measures were obtained at baseline, immediately after taping and 10 to 15 minutes after ending the duathlon competition. The kinesio tape was applied on the calf of duathletes 20 to 90 minutes before the competition, only on one of their legs (experimental leg) with the other leg acting as a control (control leg) in a randomized order. According to the between-group comparison, no differences were found immediately after the application of the kinesio tape and after the competition in the ankle range of motion and calf pain. However, a significant difference from baseline to immediately after taping was found in the ankle range of motion in the experimental leg. Applying the kinesio tape on the calf seems to immediately increase ankle dorsiflexion range of motion, but not after a duathlon competition. Applying the kinesio tape on the calf does not reduce muscle pain immediately or after a duathlon competition, but it appears to control an increase in pain.

## Introduction

The kinesio tape (KT) is a taping technique that nowadays is frequently applied in pathologies in the musculoskeletal system, especially in the field of sports injuries ( Zajt-Kwiatkowska et al., 2007 ). The unique qualities of the KT method may have multiple uses in sports injury prevention and likewise in performance ( O’Sullivan and Bird, 2011 ; Vithoulk et al., 2010 ). It can be applied theoretically to any muscle or joint of the body, and it can be worn up to four days without interfering with the daily hygiene and without modifying its adhesive properties ( Kase et al., 2003 ). The elimination of perspiration and freedom of motion are special KT characteristics that athletes appreciate ( Huang et al., 2011 ).

Kase et al. (2003) proposed several taping mechanisms with various intended outcomes depending on how the tape was applied. Using these mechanisms, different beneficial effects could be achieved, including: (1) increasing proprioception, (2) normalizing muscle tension, (3) creating more space for improving circulation, (4) correcting muscle functioning by strengthening muscle weakness, and (5) decreasing pain. Unfortunately, the limited research on the purported benefits of the KT has yielded contradictory results ( Garcia-Muro et al., 2010 ; Kaya et al., 2011 ; Paoloni et al., 2011 ; Thelen et al., 2008 ).

Duathlon is a popular sports discipline that combines running, cycling and running in one event. Ankle mobility is essential for proper running technique, especially when pushing off ( Cejuela et al., 2007 ). During duathlon competitions it is quite common to experience soreness and cramping in the calf muscles due to overuse ( Merino-Marban et al., 2011 ).

The fascia is a connective tissue that surrounds and covers muscles, which increases its tension in response to the mechanical load applied to the tissue during exercise ( O’Sullivan and Bird, 2011 ; Schleip et al., 2010 ). One theory suggests that the KT could improve sports performance by unloading the fascia, thereby relieving pain, by reducing the mechanical load on free nerve endings within the fascia ( O’Sullivan and Bird, 2011 ; Schleip et al., 2010 ).

Research based on samples of healthy athletes in order to test the effect of the KT on some aspect of performance are scarce and contradictory, and all conducted in laboratory settings ( Briem et al., 2011 ; Chang et al., 2010 ; Fu et al., 2008 ). To our knowledge, no randomized controlled research examining the effects of the KT on calf pain and ankle range of motion during competition has been carried out. Consequently, the purpose of this study was to examine the effect of the KT on calf pain and ankle dorsiflexion in duathletes immediately after its application and after a duathlon competition.

## Material and Methods

### Participants

A sample of 28 duathletes (6 females and 22 males) (age 29.11 ± 10.35 years; body height 172.57 ± 6.17 cm; body mass 66.63 ± 9.01 kg; body mass index 22.29 ± 2.00 kg/m ^2^ ) were recruited from the competitors in a duathlon sprint (5 km running + 20 km cycling + 2.5 km running). The participants were recreational duathletes involved in regular training and competition (mean training 15.59 ± 6.56 hours per week, mean competition experience 6.41 ± 6.47 years). The following inclusion criteria were used: (a) participants who are 18 years of age or older, (b) participants with more than one year of experience in a systematic duathlon training and competition, and (c) participants without any form of musculoskeletal disorder. Participants were thoroughly informed of the protocols and procedures before their participation, and written informed consent was obtained from them. The study was approved by the Ethics and Research Committee of the University of Malaga.

### Measures

*Calf pain* . The Numerical Pain Rating Scale (NPRS) (0 - no pain; 10 - maximum pain) was used to record the duathlete’s perceived level of calf muscle pain. Each participant was asked to rate their current level of pain. The participant was shown a line drawn on a sheet with “No pain” marked at one end of the scale and “Maximum pain” marked at the other. The NPRS has shown to be a reliable, valid and sensitive tool to assess pain ( Williamson and Hoggart, 2005 ).

**Figure 1:**
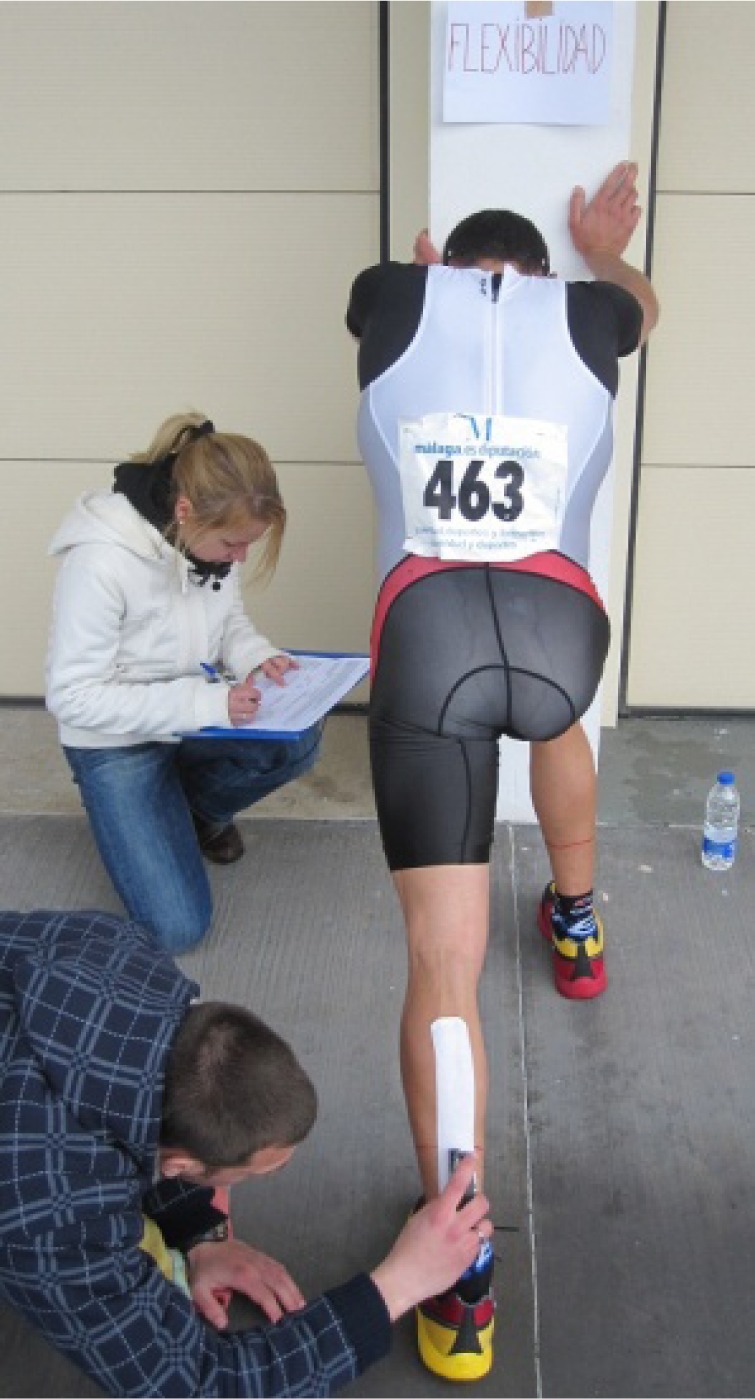
*
The range of motion measurement with the kinesio tape applied.
*

*Ankle range of motion* . Ankle dorsiflexion ROM was measured in the weight-bearing position using an inclinometer (AcuAngle®, Japan) ( Figure 1 ). This was accomplished by placing the device on the posterior aspect of the calf while the participant was standing erect, so that the calf muscle was at a 90º angle to the floor. To insure consistent placement of the inclinometer, a semipermanent mark was drawn on each participant’s calf muscle and used throughout the study. While maintaining the knee in full extension and the foot flat on the ground, the participant shifted the body over the foot as far as possible while the needle on the inclinometer moved to measure maximal dorsiflexion ROM ( Draper et al., 1998 ). All ROM measurements were collected by the same investigator. The average of two attempts was retained.

### Procedures

The present research took place on March 6, 2011, the day of the celebration of the IX Duathlon at Ronda (Malaga, Spain). Although the competition began at 10:00 am, the distribution of bib numbers started 2 hours before. While the athletes were in line waiting to pick up their bib number, they were fully informed about the purpose and procedures of the study. Out of the 141 duathletes taking part in the competition, 28 volunteered to participate in the research.

The NPRS and ankle dorsiflexion ROM were obtained at baseline (preKT), immediately after taping (postKT) and 10 to 15 minutes after finishing the duathlon competition (postRace). A 5 cm wide KT (Kinesiology tape®, Korea) was applied on the calf of duathletes between 20 to 90 minutes before the competition. The KT was applied randomly only on one leg of every duathlete (experimental leg, EL) while the other leg acted as a control (control leg, CL).

The KT was applied to the participants’ calf using the I-shaped taping technique ( Kase et al., 2003 ). While the participant was in a neutral body position, the base of the tape was placed unstretched just distal to the insertion of the muscle. Then a functional strip was applied on the stretched muscle belly, maintaining the original 10% tape pre-stretching. Afterward, the anchorage was attached unstretched, just proximal to the insertion of muscle in a neutral body position ( Kase et al., 2003 ). All KT procedures were performed by the same investigator.

### Analysis

Descriptive statistics (means and standard deviations) of body height, body mass, body mass index, training hours per week, years of competition, and the results obtained in the NPRS as well as the ankle ROM were calculated. A two-way analysis of variance (ANOVA), with the leg (EL, CL) as a between-group factor and time (preKT, postKT, postRace) as a repeated measures factor, was applied over the ankle ROM scores. For post hoc analysis, α values were corrected using the Bonferroni adjustment. As the results obtained in the NPRS did not follow a normal distribution, a non-parametric statistic was applied. The Mann-Whitney U test for between-group analysis (EL, CL) and the Wilcoxon test for within-group analysis (preKT, postKT, postRace) on the NPRS scores were used. The Hedges’ g effect size was used to determine the magnitude of treatment effects ( Hedges, 2008 ). Statistical analysis was performed using SPSS 15.0 for Windows (SPSS ® Inc., Chicago, IL). The level of statistical significance for parametric tests was set at p < 0.05 and for non-parametric tests at p < 0.0167.

## Results

Mean values and standard deviations obtained in the ankle ROM, as well as the results of repeated measures ANOVA and the Bonferroni adjustment, are presented in Table 1 . The results of the ANOVA on the average obtained in the ankle ROM showed no interaction effects between the leg (EL, CL) and time variables (preKT, postKT, postRace) [F(2, 80) = 0.154; p = 0.221]. However, for within-group analysis of EL, the ANOVA with Bonferroni adjustment showed statistically significant differences from preKT to postKT (p = 0,008). For all other comparisons of the EL, and all of the CL, the ANOVA with Bonferroni adjustment did not show significant differences.

Mean values and standard deviations obtained in the NPRS, as well as the results of the Mann-Whitney U and Wilcoxon tests, are presented in Table 2 . The results of the Mann-Whitney U tests on the average obtained in the NPRS did not show statistically significant differences between-groups (preKT, p = 0.583; postKT, p = 0.823; postRace, p = 0.882). Nevertheless, the within-group analysis with the Wilcoxon test for both legs showed statistically significant differences from postKT to postRace (EL and CL, p < 0.001) and from preKT to postRace (EL and CL, p < 0.001).

## Discussion

The purpose of this study was to examine the effect of the KT on calf pain and extensibility in duathletes immediately after its application and after a duathlon competition. According to the between-group comparison, no differences were found after the application of the KT on the ankle ROM and calf pain immediately and after the competition. However, for within-group analysis a significant difference from preKT to postKT was found in the ankle ROM in the EL, but not in the CL. On the other hand, although there were no statistically significant differences between baseline and postKT or postRace pain values, the KT in the EL seems to contribute to a better control in muscle pain after performing a duathlon competition when compared to CL ( *g* = −0.39 ).

### Ankle range of motion

The present study found a significant effect of the KT on the ankle ROM immediately after its application but not after the completion of a duathlon competition. There are two theories that may explain how the KT improves ROM. One theory posits that the KT increases blood circulation in the taped area ( Kase et al., 2003 ) and that this physiological change may affect the muscle and myofascial functions after the application of the KT. Another theory suggests that the KT stimulates cutaneous mechanoreceptors at the taped area, and this stimulation may affect the ROM ( Halseth et al., 2004 ; Murray and Husk, 2001 ). Therefore, muscle function could be improved with the KT by regulating muscle tone ( Sijmonsma, 2007 ).

In this line, previous controlled studies demonstrated an influence of the KT on the ROM immediately after its application. Thelen et al. (2008) found that the KT immediately improved the patients’ pain-free shoulder ROM. Yoshida and Kahanov (2007) observed a significant increase in active lower back flexion ROM in 30 healthy university students after KT application on the lower back. Gonzalez-Iglesias et al. (2009) found that patients with acute whiplash exhibited statistically significant improvements in cervical ROM immediately following application of the KT.

On the other hand, Merino-Marban et al. (2011) studied the acute effect of the KT on the extensibility of the hamstring muscle among 43 university students. All participants had both legs tested under three different randomly ordered conditions (KT, placebo tape and control) using the passive straight leg raise test. These authors did not observe statistically significant differences among the three study conditions. Similar results were found by Salvat and Alonso (2010) who studied the acute effect of the KT on trunk flexion. The 33 participants in their study were randomly distributed into three groups: KT, conventional tape, and placebo tape. No statistically significant differences were found among the groups on the sit and reach scores.

According to Cejuela et al. (2007) , ankle mobility is essential for an adequate reaction response when running. The tightness of the calf muscle directly influences ankle mobility ( Radford et al., 2006 ), so it could be beneficial for duathletes to maintain triceps surae extensibility, and theoretically the KT could helps along these lines.

The results of the different studies on the acute effect of the KT on the ROM are scarce and contradictory. In addition, no study was found on the effects of the KT in the ROM after performing sports competition as in the present study. Further research is needed on the effects of the KT on sports performance.

### Calf pain

The between-group comparison did not find differences after the application of the KT on calf pain immediately and after the competition. However, the within-group analysis for both legs showed statistically significant differences from postKT to postRace and from preKT to postRace. After the competition, duathletes marked statistically greater levels of calf pain in both legs than before (from postKT to postRace). Nevertheless, increased pain seemed to be considerably lower in the EL ( *g* = −0.39 ). In line with these results, some previous studies have demonstrated no statistically significant effect of the KT on pain. In the Thelon’s et al. (2008) study, the KT had no effect on pain in college students with shoulder pain.

There were no changes in pain when Firth et al. (2010) applied the KT to the Achilles tendon in 26 healthy people and 29 people with Achilles tendinopathy. Garcia-Muro et al. (2010) in a case report document the results achieved with the KT as the exclusive therapeutic procedure for the treatment of a patient with shoulder pain of myofascial origin. It is highly significant that intensity values of pain, either subjective (Visual Analog Scale) or objective (algometry), did not change between the two first measurements.

On the contrary, the positive effect of the KT in pain reduction can be observed in different studies. Kaya et al. (2011) compared the KT to physical therapy modalities in 55 patients with shoulder impingement syndrome. The rest, night and movement median pain scores of the KT group were significantly lower at the first week examination when compared with the physical therapy group. Thus, they recommend the KT especially when an immediate effect is needed. Gonzalez-Iglesias et al. (2009) demonstrated that patients with acute whiplash who received the KT exhibited statistically significantly greater improvements in neck pain immediately following the application of the KT evaluated by an 11-point numerical pain rating scale. When Paoloni et al. (2011) applied the KT to 39 chronic lower back pain patients, the pain was relieved shortly after its application. Hence, these authors recommended the KT for immediate and acute pain control.

The results of the studies presented above are contradictory, and samples formed by injured participants. Only three studies have been found on the effects of the KT on muscle pain caused by physical exercise on healthy participants, however, different outcomes were reported. Shoger et al. (2000) investigated the effect of the KT on pain using a delayed onset muscle soreness (DOMS) model in the wrist flexors. They found that the KT does not reduce the pain associated with DOMS. On the contrary, Nosaka (1999) applied an eccentric exercise to the brachium flexor group in order to cause DOMS. Maximal isometric force, range of motion, pain and plasma level of creatine kinase measurements demonstrated a tendency that the KT controlled the muscle damage and assisted in the recovery. Merino Marban et al. (2011) studied calf pain after duathlon competitions, and found a perceived pain ranging from zero to two among triathletes. These pilot studies show conflicting conclusions and highlight the need for more rigorous studies on larger sample groups. A recent theory argues that the KT could unload the fascia and thereby relief pain reducing the mechanical load on free nerve endings within the fascia ( O’Sullivan and Bird, 2011 ; Schleip et al., 2010 ).

In conclusion, applying the KT on calf seems to significantly increase ankle dorsiflexion ROM immediately, but not after a duathlon competition. Placing the KT on the calf does not seem to reduce muscle pain immediately after its application; however, it appears to control an increase in pain of triceps surae after a duathlon competition. Therefore, athletic trainers and physical therapists may apply the KT to athletes before competition in order to control soreness or cramping in the muscles.

## Figures and Tables

**Table 1:** *
Effects of the kinesio tape on the ankle range of motion (°)
*

Leg	PreKT (1) (M ± SD)	PostKT (2) (M ± SD)	PostRace (3) (M ± SD)	p ^ a ^	* Post hoc *			Effect size		

					1–2	2–3	1–3	1–2	2–3	1–3
Experimental	37.14 ± 6.44	39.76 ± 7.07	38.88 ± 6.27	> 0,05	**	-	-	0.09	−0.18	−0.09
Control	37.04 ± 6.75	39.04 ± 6.88	39.38 ± 6.68		-	-	-			

*PreKT, baseline measure; PostKT, immediately after taping measure;*

*PostRace, after finishing competition measure; M, mean; SD, standard deviation.*

p ^a^ , level of significance of the ANOVA; post hoc analysis with Bonferroni adjustement (− p > 0.05; ** p < 0.01).

**Table 2:** *
Effects of kinesio tape on calf pain
*

Leg	PreKT (1) (M ± SD)	PostKT (2) (M ± SD)	PostRace (3) (M ± SD)	p ^ a ^	* Post hoc *			Effect size		

					1–2	2–3	1–3	1–2	2–3	1–3
Experimental	1.42 ± 1.53	0.97 ± 1.20	2.32 ± 2.03	> 0,05	-	***	***	−0.07	−0.39	−0.38
Control	1.32 ± 1.53	0.98 ± 1.24	2.80 ± 2.27		-	***	***			

*PreKT, baseline measure; PostKT, immediately after taping measure;*

*PostRace, after finishing competition measure; M, mean; SD, standard deviation.*

p ^a^ , level of significance of the Mann-Whitney U test; comparison within-group analysis with the Wilcoxon test (− p > 0.0167; *** p < 0.001).
